# Fall-related injuries in a nursing home setting: is polypharmacy a risk factor?

**DOI:** 10.1186/1472-6963-9-228

**Published:** 2009-12-11

**Authors:** Federico Baranzini, Marcello Diurni, Francesca Ceccon, Nicola Poloni, Sara Cazzamalli, Chiara Costantini, Cristiano Colli, Laura Greco, Camilla Callegari

**Affiliations:** 1 Dipartimento di Medicina Clinica, Università degli Studi dell'Insubria, Via O Rossi 9, 21100 Varese, Italy; 2 Residenza Sanitaria Assistenziale (RSA) Fondazione Fratelli Molina, V le Borri, 21100 Varese, Italy

## Abstract

**Background:**

Polypharmacy is regarded as an important risk factor for fallingand several studies and meta-analyses have shown an increased fall risk in users of diuretics, type 1a antiarrhythmics, digoxin and psychotropic agents. In particular, recent evidence has shown that fall risk is associated with the use of polypharmacy regimens that include at least one established fall risk-increasing drug, rather than with polypharmacy *per se*. We studied the role of polypharmacy and the role of well-known fall risk-increasing drugs on the incidence of injurious falls.

**Methods:**

A retrospective observational study was carried out in a population of elderly nursing home residents. An unmatched, post-stratification design for age class, gender and length of stay was adopted. In all, 695 falls were recorded in 293 residents.

**Results:**

221 residents (75.4%) were female and 72 (24.6%) male, and 133 (45.4%) were recurrent fallers. 152 residents sustained no injuries when they fell, whereas injuries were sustained by 141: minor in 95 (67.4%) and major in 46 (32.6%). Only fall dynamics (p = 0.013) and drugs interaction between antiarrhythmic or antiparkinson class and polypharmacy regimen (≥7 medications) seem to represent a risk association for injuries (p = 0.024; OR = 4.4; CI 95% 1.21 - 15.36).

**Conclusion:**

This work reinforces the importance of routine medication reviews, especially in residents exposed to polypharmacy regimens that include antiarrhythmics or antiparkinson drugs, in order to reduce the risk of fall-related injuries during nursing home stays.

## Background

Around 30% of people aged 65 years or older living in the community and more than 50% of those living in residential care facilities or nursing homes fall every year and about 50% of these fall repeatedly. Nursing home residents older than 65 years of age and recording a rate of falls (falls/bed/year) of 1.5 are approximately three times more likely to fall than their community-dwelling peers [1].

Falls often lead to reduced functioning, which increases morbidity and mortality: around 20% of falls need medical attention, 5% result in fractures, severe head injuries, joint distortions and dislocations, and 5-10% in soft-tissue contusions and lacerations. Fall-induced injuries are the fifth leading cause of death in elderly adults and are one of the most common causes of longstanding pain and disability in this population: falls account for over 80% of injury-related admissions to hospital of people older than 65 years [2]. Between 10% and 25% of nursing home falls result in fractures or hospital admissions [1].

The risk of falling increases dramatically with a number of risk factors, such as musculoskeletal problems, neurological diseases, psychosocial characteristics, functional dependency and drug use. Prevention is not easy because falls are complex events caused by a combination of intrinsic impairments and disabilities and, sometimes, environmental hazards [3].

Polypharmacy is regarded as an important risk factor for falling and several studies and meta-analyses have shown an increased fall risk in users of diuretics, type 1a antiarrhythmics, digoxin and psychotropic agents [4,5]. A randomised trial confirmed that gradual withdrawal of psychotropic drugs reduces the risk of falling by 66% [6]. Polypharmacy is quite a widespread practice in many clinical settings, including nursing homes. Although there still exists no clear and universally accepted definition of polypharmacy, it is known to be related to the onset of drug-related problems [7]. Polypharmacy is often associated with pointless or inappropriate prescriptions that increase the likelihood of patients, particularly the elderly, manifesting sedation, confusion, balance disorders and complications caused by pharmacological interactions [8].

In particular, there is recent evidence that fall risk is associated with the use of polypharmacy regimens that include at least one established fall risk-increasing drug, rather than with polypharmacy *per se *[9]. Several studies have demonstrated a direct correlation between psychotropic medication use, polypharmacy and fall rate. However, no clear relationship between psychotropic drugs and fall-related injuries, in particular fractures, has been demonstrated.

In the light of the international evidence, we studied the role of polypharmacy and the role of well-known fall risk-increasing drugs on the incidence of injurious falls. Our aim was to demonstrate that the association between polypharmacy and injuries is explained by the higher probability, in a polypharmacy regimen, of receiving a fall risk-increasing drug. To test this hypothesis, we assessed the association between polypharmacy and injuries in a nursing home setting in the North of Italy.

## Methods

This retrospective observational study was carried out from July 1st, 2004 to December 31^st^, 2007 at the "*Residenza Sanitaria Assistenziale (RSA) Fondazione Fratelli Molina*" in Varese (Italy), a state-funded nursing home providing assistance and rehabilitation for elderly people who, for the most part, are no longer self-sufficient. The *Fondazione Fratelli Molina *nursing home is composed of four large buildings, or units, and it has about 450 beds. The units are heterogeneous as regards their architectural design, care protocols and number of beds. The home is staffed by physicians specialising in internal medicine and geriatrics, registered nurses, physiotherapists, psychomotor therapists, care assistants, social workers and occupational therapists. Since March 2000, a consultation-liaison project has been in place between this institution and the Psychiatry Unit of the Department of Clinical Medicine of the University of Insubria in Varese.

Of the 1198 residents in the nursing home in the course of the study period, fall injury event data were recorded, during that time, for 293, aged between 65 and 101 years. An unmatched, post-stratification design for age, gender and length of stay was adopted [10]. We excluded residents who were bedbound, bilateral amputees [11], non-Italian speakers, affected by Alzheimer's disease or attending the day care rehabilitation departments, and those who reported falls as a result of altercations.

For each patient we collected the following data:

▪ Socio-demographic and clinical data: age, gender, date of admission to the home, number of psychiatric consultations received, level of disability as recorded in the patient's notes at the time of the first fall injury event (totally non-self-sufficient or self-sufficient, the latter referring to residents scoring 100 on the Barthel Index), and psychiatric diagnosis made by the consultant specialist in psychiatry. Particular attention was paid to the presence of psycho-organic disorders (i.e., psychiatric disorders secondary to organic brain disease, brain injury, or other insult leading to cerebral dysfunction). We also noted which of the four units the patient resided in, although no unit characteristics were gathered.

▪ Fall-related data: data referring to the first fall injury event were gathered from the register of falls and injuries in which reports are usually entered by nurses who can request medical advice should a patient's clinical conditions warrant it. These reports contain: fall date, fall dynamics (classified as: a slip on the floor, an attempt to stand up, a fall from the wheelchair, a fall in the bedroom and a "random" or incidental fall, when not otherwise explained) and fall outcome (severity of injuries). Fall-related injuries were classified as: minor, such as bruising, sprains, cuts, abrasions (injuries that required a nurse's intervention or local medication) or major, such as fractures, distortions, skin lacerations requiring suturing, loss of consciousness (injuries that required a doctor's intervention and medication, diagnostic investigation, an emergency department visit or hospitalisation) [12]. In accordance with the literature definition, a "fall" was taken to mean "a sudden and unintentional change of position, with or without loss of consciousness, causing the victim to land on the ground" [13]. Recurrent fallers were individuals who "reported two or more falls" in one year [14,15].

▪ Pharmacological data: we recorded details of all the medications the patient was taking at the time of the fall, including sleeping tablets (taken as necessary). However, we did not consider local topical and ophthalmological drugs since they have minimal systemic effects, or antibiotics, given their lack of effect on the central nervous system. In accordance with the WHO Anatomical Therapeutic Chemical (ATC) classification system [16], psychotropics were categorised as: typical antipsychotics (psycholeptics ATC N05A, excluding atypical antipsychotics) and atypical antipsychotics (clozapine, aripiprazole, risperidone, olanzapine, quetiapine), benzodiazepines (anxiolytics, benzodiazepine derivates ATC N05BA), hypnotics and sedatives (ATC N05CD and N05CF), antidepressants (ATC N06A, excluding new generation ones), new generation antidepressants (selective serotonin reuptake inhibitors, selective noradrenergic reuptake inhibitors, or combined action antidepressants) and antiepileptics (ATC N03). Other medications considered were: antihistamines (ATC R06A), antihypertensives/diuretics (ATC C02, C03), antiarrhythmics (ATC C01B), antiparkinson drugs (ATC N04), vitamins (ATC A11), blood glucose lowering drugs and insulin (A10A, A10B), opioid analgesics (ATC N02A), antithrombotic agents (ATC B01A), non-steroidal anti-inflammatory drugs (NSAIDs) (ATC M01A), cardiac glycosides and nitrates (ATC C01A, C01DA), and gastrointestinal agents (ATC A03). Even though there is no clear consensus in the literature, polypharmacy was defined as "the use of four or more medications" [4,5].

Patient frailty was assessed every three months by a resident doctor. For the purposes of our study, we considered the last evaluation before the recorded fall:

▪ The Barthel Index [17]: the patient's level of self-sufficiency was ascertained using the functional evaluation form of the Barthel Index, which evaluates movement performance and personal autonomy, taking into consideration bed-chair transfers, ambulation, wheelchair transfers, personal hygiene and the ability to feed oneself. A "self-sufficient" patient was defined as one scoring 100 on the Barthel Index.

▪ The GBS (Gottfries-Brane-Steen) scale for dementia [18]: using this instrument, the patient's mental state and behaviour were assessed taking into consideration the possible presence of derangement, irritability and restlessness. The GBS scale is divided into four subscales measuring motor, intellectual and emotional functions and different symptoms characteristic of dementia. The scale can be used by physicians, psychologists and registered nurses.

▪ The 14-item Cumulative Illness Rating Scale (CIRS) [19]: the CIRS was used to assess comorbid somatic diseases, namely cardiac, blood pressure, vascular system, respiratory system, ophthalmological and otorhinolaryngological, gastrointenstinal, hepatic, renal, urinary tract, muscular, neurological (e.g. Parkinson's disease, multiple sclerosis, amyotrophic lateral sclerosis), metabolic and psychiatric conditions. For each subscale, a dichotomous index (high or low impairment) was used.

▪ The Mini Mental State Examination (cut-off: <24 points) [20] or the Short Portable Mental Status Questionnaire (cut-off: >2 points) [21]: these instruments were used to evaluate cognitive impairment.

The study was approved by the institutional review board of the *Fondazione Fratelli Molina*.

### Analysis

Age, gender, status as a recurrent faller, known risk factors for falling, and index of comorbid conditions (CIRS) were added to a preliminary chi-square test to weigh their association with the dependent variable (fall with or without injuries). The association between risk factors and fall-related injuries was analysed using the two-sided Pearson chi-square test (p value < 0.05) and the bivariate correlation procedure (Spearman's rho coefficient) for the unadjusted univariate analysis. Then a correlational analysis was conducted among selected factors to avoid residual multicolinearity. If significant correlation was found between independent variables, these variables were omitted from the model or combined as appropriate. Moreover, interaction terms between medications were also investigated at this stage. In particular, possible interactions between each single drug category (Table 1) and polypharmacy were investigated. This was done both considering polypharmacy as "the use of four or more medications" and also taking into account an incremental definition of polypharmacy, up to "seven or more" medications.

**Table 1:** Medication use among patients

Characteristics of fallers	Injured	Not injured			
	**141 (48.1%)**	**152 (51.9%)**	p*	**O.R.**	**95% C.I.**

No. of medications			0.50		
0	1 *(0.7%)*	0 *(0%)*		ref	--
1-3	26 *(18.4%)*	32 *(21.1%)*		0.86	0.48-1.56
4 or more	114 *(80.9%)*	120 *(78.9%)*		0.96	0.25-3.71

No. of psychotropic medications			0.88		
0	28 *(19.9%)*	27 *(17.8%)*		ref	--
1-3	108 *(76.6%)*	120 *(78.9%)*		0.92	0.41-2.06
4 or more	5 *(3.5%)*	5 *(3.3%)*		1.27	0.23-7.03
Typical antipsychotics	34 *(24.1%)*	42 *(27.6%)*	0.49	0.83	0.49-1.40
Atypical antipsychotics	9 *(6.4%)*	19 *(12.5%)*	0.07	0.47	0.20-1.09
Antidepressants	4 *(2.8%)*	2 *(1.3%)*	0.34	2.19	0.39-12.14
New generation antidepressants	31 *(22.0%)*	35 *(23.0%)*	0.83	0.94	0.54-1.63
Benzodiazepines	78 *(56.0%)*	90 *(58.6%)*	0.66	0.90	0.56-1.43
Hypnotics & sedatives	3 *(2.1%)*	3 *(2.0%)*	0.92	1.08	0.21-5.43
Antiepileptics	36 *(25.5%)*	37 *(24.3%)*	0.81	1.06	0.62-1.81
Antihistamines	5 (3.5%)	9 *(5.9%)*	0.34	0.58	0.19-1.78
Antihypertensives & diuretics	104 *(74.5%)*	106 *(69.1%)*	0.30	1.30	0.78-2.17
Antiarrhythmics	16 *(11.3%)*	13 *(8.6%)*	0.42	1.36	0.63-2.95
Hypoglycaemics & insulin	26 *(19.1%)*	27 *(17.1%)*	0.65	1.14	0.63-2.08
Antiparkinson drugs	24 *(17.0%)*	21 *(13.8%)*	0.44	1.28	0.67-2.41
Vitamins	61 *(43.3%)*	71 *(46.7%)*	0.55	0.87	0.54-1.37
Opioid analgesics	10 *(7.1%)*	9 *(5.9%)*	0.68	1.21	0.47-3.07
Antithrombotic agents	58 *(41.8%)*	71 *(46.1%)*	0.46	0.84	0.53-1.33
NSAIDs	31 *(22.0%)*	30 *(19.7%)*	0.63	1.14	0.65-2.01
Cardiac glycosides & nitrates	43 *(30.5%)*	47 *(30.9%)*	0.93	0.98	0.59-1.61
Gastrointestinal agents	78 *(56.0%)*	86 *(55.9%)*	0.93	1.01	0.63-1.59

(*) Logistic regression analysis, ENTER method;

Factors and interaction terms associated with the dependent variable were entered into the logistic regression model (enter method). Only the first fall injury event, for each resident, was included in the statistical model.

Estimates of the relative risk of being injured were expressed as odds ratios (ORs) and 95% confidence intervals (CIs).

All statistical analyses were performed using SPSS^® ^13.0 for Windows^® ^(SPSS^® ^Inc, Chicago, IL).

## Results

The register of falls and injuries contained records of a total of 695 falls involving 293 residents, 221 (75.4%) females and 72 (24.6%) males; 133 (45.4%) were recurrent fallers. One hundred and fifty-two residents were not hurt when they fell, whereas injuries were sustained by 141: minor in 95 (67.4%) and major in 46 (32.6%). The prevalence of injuries showed no relationship with age, gender, psychiatric diagnosis, being a recurrent faller, length of stay, unit of residence, or patient status at the end of the period considered (not shown in Table 2). Moreover, the proportion of residents who died during the study period was similar in the two groups: 64 (45.5%) of the injured residents and 63 (41.4%) of those who did not sustain injuries.

**Table 2:** Patient characteristics

Characteristics of fallers	Injured	Not injured			
	**141 (48.1%)**	**152 (51.9%)**	p*	**O.R.**	**95% C.I.**

Age group (years)			0.83		
65-75	18 *(12.8%)*	19 *(12.5%)*		ref	--
75-85	51 *(36.2%)*	56 *(36.8%)*		0.96	0.45-2.03
85-95	62 *(44%)*	70 *(46.1%)*		0.93	0.45-1.93
>95	10 *(7.1%)*	7 *(4.6%)*		1.50	0.47-4.81

Female	107 *(75.9%)*	114 *(75%)*	0.86	1.04	0.60-1.70

Recurrent faller	60 *(42.9%)*	73 *(47.7%)*	0.40	0.82	0.50-1.30

(*) Pearson chi-square test;

A weak correlation emerged between being a recurrent faller and sustaining major injuries (Spearman's rho = -0.130 p = 0.026). Forty residents had more than four falls during the study period, and two of these suffered 15 falls. The demographic characteristics of the 293 residents are set out in Tables 2 and 3.

**Table 3:** Patient characteristics

Characteristics of fallers	Injured	Not injured	
	**141 (48.1%)**	**152 (51.9%)**	p*

Mean age in years (+/- Stand Dev)	84.6 ± 8.2	84.8 ± 7.7	0.80

No. of psychiatric consultations/person/year	1.18	1.19	-

Length of stay (± SD) in months	32.5 ± 57.5	29.6 ± 48.7	0.63

Falls/person/year	0.60	0.77	-

Mean no. of somatic co-morbidities (± SD)	9.96 ± 3.15	9.79 ± 3.19	0.65

(*) Student's t-test

Some fall dynamics were significantly associated with injuries (df = 14; p = 0.033; not shown in the tables) namely (injured - not injured): slip on the floor (3.6% vs 7.8%), attempt to stand up (12.3% vs 4.6%), fall from a wheelchair (7.2% vs 11.8%), fall in the bedroom (8.6% vs 17%) and random fall (23.7% vs 12.4%).

Of the 293 fallers, 18 (6.1%) suffered bone fractures. Of the residents who were injured when they fell, 30 (21.6%) were taken to the general hospital emergency department for further checks.

The prevalence of injuries was not related to the mean number of somatic comorbidities or psychiatric diagnoses, or to the number of self-sufficient residents. No significant differences emerged when considering the single items of the Barthel Index, the GBS scale, or the CIRS (data not shown in the tables).

Fall data and clinical characteristics of the residents are given in Table 4.

**Table 4:** Falls and clinical characteristics

Characteristics of fallers	Injured	Not injured			
	**141 (48.1%)**	**152 (51.9%)**	p*	**O.R.**	**95% C.I.**

No. of falls			0.046		
1	79 *(56%)*	78 *(51.3%)*		Ref.	--
2 -- 4	50 *(35.5%)*	46 *(30.3%)*		1.07	0.64-1.78
> 4	12 *(8.5%)*	28 *(18.4%)*		0.42	0.20-0.89

Psychiatric diagnosis^1^			0.42		
None	25 *(17.7%)*	20 *(13.2%)*		Ref.	--
Anxiety d.	13 *(9.2%)*	16 *(10.5%)*		0.65	0.25-1.66
Depressive d.	11 *(7.8%)*	27 *(17.8%)*		0.32	0.13-0.81
Schizophrenic d.	4 *(2.8%)*	4 *(2.6%)*		0.80	0.18-3.60
Bipolar mood d.	12 *(8.5%)*	14 *(9.2%)*		0.68	0.26-1.80
Psycho-organic d.	4 *(2.8%)*	2 *(1.3%)*		1.60	0.26-9.64
Cognitive impairment	54 *(38.2%)*	52 *(34.2%)*		0.83	0.41-1.67
Anxious-depressive d.	16 *(11.3%)*	15 *(9.9%)*		0.85	0.34-2.13
Personality d.	2 *(1.4%)*	2 *(1.3%)*		0.80	0.10-6.19

Self-sufficient ^2^	64 *(45.4%)*	62 *(41.1%)*	0.45		

(*) Logistic regression analysis, ENTER method; (^1^) Psychiatric Diagnosis: d. = disorder; (^2^) A "self-sufficient" patient was defined as scoring 100 on the Barthel Index

The prevalence of injuries was not found to be related to intake of increasing numbers of medications or to the use of psychotropic medications. Table 1 details the residents' use of the different medication classes.

Almost all the sample (99.7%, n = 292) were taking at least one drug and 79.9% (n = 234) were taking four or more (16 in one case). The mean intake was between five and six medications, while both the mode and the median were five; 81.2% (n = 238) of the residents were taking at least one psychotropic drug and only 3.4% (n = 10) were taking four or more psychotropic drugs (six in one case). The mean number of psychotropic medications taken was between one and two. The probability of using a psychotropic drug increased with increases in the total number of medications taken, being 0.3% in residents with only one prescription, 3.4% in those with two prescriptions, 7.2% in residents with three prescriptions, and 70.3% when four or more drugs were prescribed.

**Figure 1:**
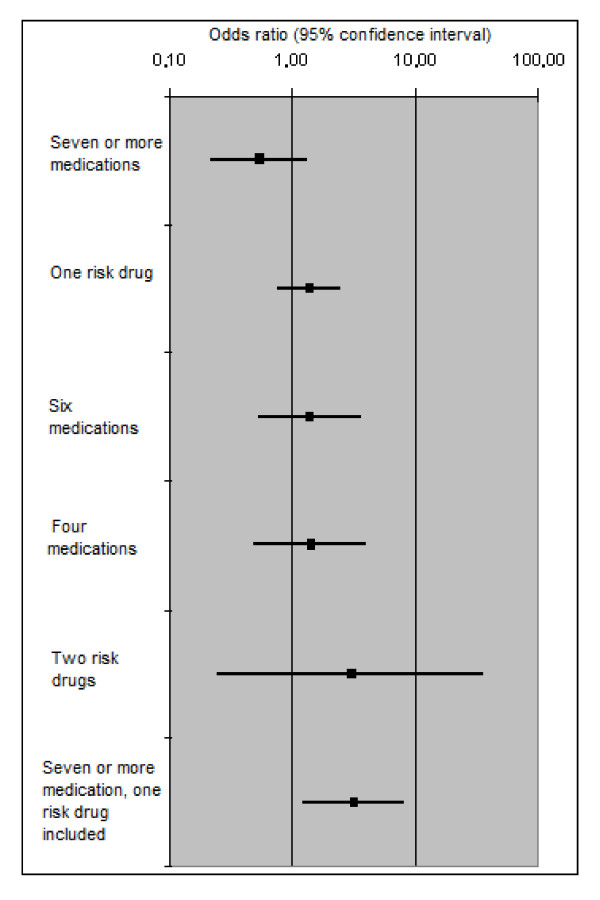
**Influence of medication combinations on the OR for an injurious fall event compared with a fall with no injury**.

On the basis of the OR values reported in Table 1, no one medication category could be considered significantly associated with the dependent variable. As regards the interactions, only taking an antiarrhythmic or an antiparkinson drug as part of a regimen of seven or more medications was significantly associated with a three-fold increased risk of reporting a fall-related injury (p = 0.017 O.R. = 3.11 95% C.I. = 1.22-7.89) after adjusting for age, gender and number of medications (Figure 1). These drugs were considered "risk drugs".

The probability of using a "risk drug" increased proportionally with increases in the total number of medications taken, from 12% with the use of three prescriptions to 39.5% when eight or more drugs were prescribed (43 residents, 14.7% of the sample).

In a logistic regression model fully adjusted for age, gender, comorbid conditions (CIRS), cognitive status (GBS), degree of mobility (Barthel Index), fall dynamics, psychiatric diagnosis, length of stay, disability (self-sufficient or not) and unit of residence (292 cases; chi-square model = 111.337, df = 90 p = 0.028; Nagelkerke R Square = 0.44; Hosmer and Lemeshow Test = 0.353; Overall Percentage = 74.3), injuries showed an association with fall dynamics (p = 0.013), in particular with incidental or random falls (p = 0.006; OR = 1.82; CI 95% 1.18-2.81) when compared with "an attempt to stand up". Recurrent faller status, as defined above, did not show an association with the dependent variable (p = 0.056; OR = 0.67; CI 95% 0.45-1.01) or with depressive disorder (p = 0.128; OR = 0.38; CI 95% 0.11-1.31). In this model, the interaction term (taking antiarrhythmic or antiparkinson drugs and using a total of seven or more medications) showed an increased OR for suffering a fall-related injury (p = 0.024; OR = 4.4; CI 95% 1.21-15.36).

## Discussion

In this nursing home study, polypharmacy *per se *was not found to be a risk factor for fall-related injuries: injuries were associated with the use of multiple drugs (7 or more), but only when an injurious fall risk-increasing drug (antiarrhythmic or antiparkinson drug) was part of the patient's therapeutic regimen. Multiple medications, or particular medication classes, were not clearly associated with injurious falls. Several literature reports support the hypothesis that these active principles can contribute to predisposing patients to falls. In particular, a meta-analysis study showed digoxin, type 1a antiarrhythmic and diuretic use to be associated with falls in older adults [5], while in another study, hip-fracture patients, compared with matched controls, were more frequently prescribed antiparkinson drugs [22]. Polypharmacy *per se*, arbitrarily defined as the use of four or more medications, was recently shown not to be an independent risk factor for falls in a population-based setting [9] and for the onset of drug-related problems [7]. Polypharmacy has been discussed extensively and at length in the scientific literature, but the concept still lacks an unequivocal and clearly accepted definition. Recent attempts to establish a cut-off value in drug intake (number of drugs used), which might make it easier to identify patients at risk, have proved unsuccessful [7,23]. Even though a relationship between the number of drugs taken and the occurrence of drug-related problems has been demonstrated, such a relationship may not be universally valid and must always be considered in the context of the specific clinical setting and the peculiarities of the population considered, in this case the frail institutionalised elderly.

The demographic characteristics of the sample included in this study appear to reflect what is known about nursing home populations: the subjects had a very advanced mean age and comorbidities were common, as was absence of self-sufficiency. In short, they showed the clinical profile typical of frail, very elderly people [11].

Another finding supported by literature data was the frequency of major injuries sustained by our residents as a result of falls. The rates of fractures and emergency department referrals reported in our sample are consistent with review data [1] and the prevalence of fractures and hospitalisations also seemed to be in line with literature data, confirming that the nursing home in which this study was conducted is very similar to the other settings with which it was compared.

Nearly half of our sample were recurrent fallers. This is in line with literature data [1] referring to a wider elderly population. We were interested to discover the existence of an inverse correlation between recurrent fallers and severe injuries. It is likely that recurrent fallers are residents well known to the nursing staff of his unit and receive more attention. That last consideration could explain why, in the logistic regression model (that takes into account unit of residence), this correlation is no longer significant.

It is worth noting that the significant association between fall-related injuries and the variable "fall dynamics" persisted in the multivariate analysis. This seems to suggest that the situational dynamics, perhaps linked to the patient's clinical conditions and daily habits, are closely related to the risk of exposure to injury. To our knowledge, few studies have focused on fall dynamics, given that it is a secondary factor and difficult to classify. Thapa et al. conducted a study that, like ours, was based on internal nursing care records [24], while Chen and colleagues found that falls occurring outside were associated with major injuries (fractures) and a lower level of mobility [11]. Contrary to Chen's findings, in our sample, no differences were found between the self-sufficient and the non-self-sufficient residents.

The results of the multivariate analysis showed that fall dynamics is a variable that can significantly affect the outcome of a fall. Injuries were found to be sustained less frequently by residents who fell from wheelchairs, probably because they did not have so far to fall, a finding also confirmed by others [11], and by residents who fell while in their bedrooms, probably because the bedroom is a more familiar environment where there are more likely to be objects that residents can grab hold of to break their fall. Conversely, falling after standing up or random falls were associated, on multivariate analysis, with a significantly increased injury rate. This is probably due to the drop in blood pressure that is induced by standing up, as Thapa et al. reported in non-ambulatory nursing home residents [24], or that can be caused by unexpected circulatory failure.

In contrast to recently published data [25,26], we did not find atypical antipsychotic drug use to be associated with fall-related injuries or femur fractures. This finding could be biased by the low rate of use of this drug class in our residents (despite their extrapyramidal symptom profile), or by the absence of dose data. The US Food and Drugs Administration warning on the increasing death rate among patients using second generation antipsychotic medications has undoubtedly influenced their prescription rate in elderly demented patients with behavioural disorders (in whom off-label use of atypical antipsychotics used to be common) [27].

The majority of our sample of nursing home residents took at least one psychotropic drug. This suggests that psychotropic medications, particularly the new generation antidepressants and benzodiazepines, are well known in nursing home settings, and that they are considered manageable and safe by the medical staff.

In our study, these two psychotropic drug classes [28], well known as "fall risk medications", do not seem, *per se*, to be injury risk factors. Being a "fall risk medication" does not automatically imply that a medication is also an independent "injury risk medication", especially in a nursing home setting: other factors, such as the impact with the floor and the patient's strength and bone structure, seem to be more important in determining the outcome of a fall [11].

This observation could be attributable, in part, to the high level of comorbidity in this population. Severe ophthalmological, cardiological and otorhinolaryngological system impairment associated with cognitive-neurological impairment are important risk variables when associated with patient drug regimens. Other factors, in particular unit characteristics (environment or staffing levels) and fall dynamics or their association, seem to be very important in determining fall-related injuries.

In addition to the above mentioned high level of comorbidity, an association also emerged with the presence of a depressive disorder; these disorders are frequently underestimated in nursing homes [29]. As shown in Table 4, depressive status (compared to not having any psychiatric diagnosis), appeared to be a protective factor against fall-related injuries. However, on the multivariate analysis, in which the use of antidepressants was also considered, this protective effect was cancelled out.

This study, having a retrospective design and investigating injury rates, does present some limitations. First of all, it does not allow us to comment on fall risk factors, since the comparisons were between fallers sustaining and fallers not sustaining injuries. Second, because of the type of design adopted, we could not assume, with any degree of certainty, the existence of any temporal relationship between the event and the presence of risk factors. However, in view of the setting in which this study was conducted, it can be assumed that these patients, affected by chronic diseases, used their drugs constantly and as prescribed. Third, as in all observational studies, independent variables may be associated with unknown confounders (confounding by indication) that were not taken into account, for example, unit characteristics (environmental factors and staffing levels).

Other clear limitations include the fact that this study was underpowered to estimate the individual effect of each medication. The agents were included in the analysis grouped by categories. Another limitation is that it failed to address the questions of medication dosages, area of impact or type of floor surface where falls occurred, residents' balance impairments and bone fragility indices. These data, in addition to medications used and clinical status, would be useful in investigating the relationship between risk factors and types and sites of injuries.

## Conclusion

Polypharmacy has been linked to an increased risk of falling. We set out to investigate the extent to which polypharmacy, as usually defined, creates an increased risk of sustaining injuries in a nursing home setting.

In accordance with the literature we found an association between medication use and injuries. In particular, the main findings of our study highlight that polypharmacy is not a risk factor *per se*, but plays a relative role; they also highlight the significant role played by identifiable setting-specific risk factors for injuries (drugs and fall dynamics) in an elderly institutionalised population.

In this study, "fall dynamics", among the variables independently associated with the risk of sustaining a fall-related injury, was found to play a surprisingly prominent role. As other authors have suggested [11], this aspect would certainly be worth exploring in greater depth in the future, so as to have a more complete picture for preventive risk assessment.

In conclusion, this study, in accordance with part of the literature in this field, confirms the impossibility of establishing a specific definition of polypharmacy that might be universally accepted as an independent factor associated with the risk of sustaining fall-related injuries. This risk can really be assessed only taking into account other dimensions, like the dynamics of the fall and the association with the medications typically used by a specific group, such as the very elderly institutionalised population. All this reinforces the importance of a critical routine medication reviews to reduce the risk of fall-related injuries during nursing home stays.

## Competing interests

The authors declare that they have no competing interests.

## Authors' contributions

FB conceived of the study and participated in its design; MD partecipated in drafting and revising the manuscript; FC partecipated in drafting and revising the manuscript; NP partecipated in drafting and revising the manuscript; SC was involved in the acquisition of data and performed the statistical analysis; CC was involved in the interpretation of data and performed the statistical analysis; CC was involved in the acquisition and analysis of data; GL participated in the design and coordination of the study; CC made substantial contributions to conception and design of the study.

All authors read and approved the final manuscript.

## Pre-publication history

The pre-publication history for this paper can be accessed here:

http://www.biomedcentral.com/1472-6963/9/228/prepub
